# Methyl jasmonate ameliorates pain‐induced learning and memory impairments through regulating the expression of genes involved in neuroinflammation

**DOI:** 10.1002/brb3.3502

**Published:** 2024-04-29

**Authors:** Fatemeh Mohammadinia, Saeed Esmaeili‐Mahani, Mehdi Abbasnejad, Manijeh Dogani, Ali Mohammad Poorrahimi

**Affiliations:** ^1^ Kerman Neuroscience Research Center, Institute of Neuropharmacology Kerman University of Medical Sciences Kerman Iran; ^2^ Department of Biology, Faculty of Sciences Shahid Bahonar University of Kerman Kerman Iran

**Keywords:** CA1, capsaicin, learning and memory dysfunction, methyl jasmonate, orofacial pain

## Abstract

**Objective:**

Orofacial pain with high prevalence is one of the substantial human health issues. The importance of this matter became more apparent when it was revealed that orofacial pain, directly and indirectly, affects cognition performances. Currently, researchers have focused on investigating pharmaceutics to alleviate pain and ameliorate its subsequent cognitive impairments.

**Design:**

In this study, the rats were first treated with the central administration of methyl jasmonate (MeJA), which is an antioxidant and anti‐inflammatory bio‐compound. After 20 min, orofacial pain was induced in the rats by the injection of capsaicin in their dental pulp. Subsequently, the animals’ pain behaviors were analyzed, and the effects of pain and MeJA treatments on rats learning and memory were evaluated/compared using the Morris water maze (MWM) test. In addition, the expression of tumor necrosis factor‐α *(TNF‐α)*, *IL‐1β*, *BDNF*, and *COX‐2* genes in the rats’ hippocampus was evaluated using real‐time polymerase chain reaction.

**Results:**

Experiencing orofacial pain resulted in a significant decline in the rats learning and memory. However, the central administration of 20 μg/rat of MeJA effectively mitigated these impairments. In the MWM, the performance of the MeJA‐treated rats showed a two‐ to threefold improvement compared to the nontreated ones. Moreover, in the hippocampus of pain‐induced rats, the expression of pro‐inflammatory factors *TNF‐α*, *IL‐1β*, and *COX‐2* significantly increased, whereas the BDNF expression decreased. In contrast, MeJA downregulated the pro‐inflammatory factors and upregulated the BDNF by more than 50%.

**Conclusions:**

These findings highlight the notable antinociceptive potential of MeJA and its ability to inhibit pain‐induced learning and memory dysfunction through its anti‐inflammatory effect.

## 1 INTRODUCTION

About 15% of the adult population experience orofacial pain, which, after back, neck, and knee pain, is one of the most common chronic pains (Häggman‐Henrikson et al., 2020). Likewise, it is known as a significant clinical issue with detrimental social and economic consequences. Regarding its origin, this kind of pain originates from regions that are innervated mainly by trigeminal nerve branches (Mačianskytė et al., 2011). Dental pulp pain is one of the most common orofacial pains. It has recently been demonstrated that capsaicin (Cap)‐sensitive nerve fibers are present in dental pulp tissue. These fibers express transient receptor potential vanilloid 1 (TRPV1) channels that are triggered by various thermal and chemical stimulants, such as Cap (Rahbar et al., 2019). Activation of TRPV1, in turn, initiates some downstream events, such as enhancing cytosolic calcium influx and inducing pro‐nociceptive inflammatory mediators. Interleukin‐1β (IL‐1β), IL‐6, tumor necrosis factor‐α (TNFα), Substance P, and calcitonin gene–related peptide are among the inflammatory mediators relating to the perception or sensation of pain (Kaczmarski et al., 2022). Trigeminal nerve–related issues, in addition to sensory characteristics, are generally accompanied by unfavorable neurobehavioral subsequent consequences (Alawi & Keeble, 2010; Rahbar et al., 2019). Dental pulp pain, in this regard, is one of the most common types of orofacial pain causing adverse neurobehavioral dysfunctions, including cognitive impairments (Rafie et al., 2022).

Researchers suggest a mutual interaction between pain and cognitive function. That is, pain interferes with cognitive performance, and cognitive operations can conversely decrease pain perception (Mazza et al., 2018). Recent studies have provided evidence indicating the incidence of impaired cognitive function in individuals experiencing chronic pain (Zhang et al., 2021). It has also been revealed that pain can disturb memory acquisition, consolidation, and retention processes in rats (Shahsavari et al., 2018). The precise mechanism of action justifying these observations has not been cleared yet. However, studies have reported the enhanced release of pro‐inflammatory and pro‐apoptotic compounds in the hippocampus of rats following pain stimulation (Raoof, Esmaeili‐Mahani, Nourzadeh, et al., 2015). In addition, based on recent studies, dental pulpal pain induced by Cap caused learning and memory impairment, mainly due to the induction of apoptosis in the rats’ hippocampus neuronal cells (Raoof, Esmaeili‐Mahani, Nourzadeh, et al., 2015; Raoof, Esmaeili‐Mahani, Abbasnejad, et al., 2015). There are a bunch of studies that support the relationship between chronic pain and cognitive dysfunction, yet limited studies are paid to orofacial pain (Moriarty et al., 2011).

Recently, studies have mostly focused on finding effective agents to alleviate/ameliorate cognitive impairments and improve memory and learning performances, and fortunately, promising outcomes have been achieved (Fereidooni et al., 2020). It has been found that analgesic drugs could effectively alleviate pain‐induced dysfunctions (Sethna et al., 1998). In line with this, methyl jasmonate (MeJA), a phytohormone that was initially extracted from *Jasminum grandiflorum* L., though it can be found in various plants and some microorganisms too (Aluko et al., 2021; Reyes‐Díaz et al., 2016), has gained much attention due to its biological properties. Naturally, this compound regulates intracellular signaling mechanisms involved in the plant growth process, defense system, and response to stress stimuli. According to the literature, MeJA is known as safe and can be vastly applied in the food industry. The compound enhances the antioxidant capacity and flavonoid content of fruits (Alvarez et al., 2015; Wang et al., 2008). Jasmonic acid, *cis*‐jasmonate, and MeJA are the main members of the jasmonate family. There is a striking resemblance between the chemical structure of these compounds and prostaglandins possessing anti‐inflammatory and antioxidant activities (Sá‐Nakanishi et al., 2018). Hence, recently, some investigations have been carried out to evaluate their therapeutic effects on behavioral and neurochemical functions (Annafi et al., 2017). The effects of MeJA on cognitive functions have been previously documented (Eduviere et al., 2015; Hemati et al., 2021). However, the ability of MeJA to modulate pain‐induced learning and memory impairments has not been investigated yet.

The present study aimed to investigate the effect of the central administration of MeJA on pulpal pain, learning, and memory dysfunction in a rat model. Toward this end, the pulpal nociception was evoked by the interdental injection of Cap in the rats, and the pain behaviors in animals were evaluated and compared with the animals that received MeJA and the intact ones. We also evaluated the learning and memory performance of these animals. In addition, the expression of some genes involved in the inflammatory conditions, such as *TNF‐α*, *IL‐1β*, *BDNF*, and *COX‐2*, was evaluated in the hippocampus.

## 2 MATERIALS AND METHODS

### 2.1 Drugs

The Cap used in this study was purchased from Sigma‐Aldrich, and prior to being utilized, it was dissolved in ethanol/Tween 80/distilled water. The MeJA was also supplied with Sigma that was dissolved in physiological serum (0.9% NaCl).

### 2.2 Animals

Thirty‐six adult male Wistar rats, weighing 200 ± 20 g, were purchased from the Laboratory Animal Maintenance and Breeding Center of Kerman University of Medical Sciences and housed under controlled conditions (23 ± 1°C; 12:12 h light/dark cycle) with free access to food and water. Moreover, they have been given a week to become accustomed to the circumstances before tests.

During this study, all experiments were performed following the University of Medical Sciences and Neuroscience Ethics Committee guidelines (IR.UK.VETMED.REC.1402.003).

### 2.3 Experimental design

After 1 week of adaptation, the rats were randomly divided into six groups (*n* = 6), as follows:

1. Control: intact rats
2. Cap: The rats that received 100 μg/rat of Cap dissolved in ethanol/Tween 80/distilled water to evoke dental pulp pain
3. Vehicle: The rats that were treated with Cap and vehicle of MeJA (0.9% NaCl)
4. MeJA 5: The rats that were subjected to pain induction following 5 μg/rat of MeJA treatment
5. MeJA 10: The rats that were subjected to pain induction following 10 μg/rat of MeJA treatment
6. MeJA 20: The rats that were subjected to pain induction following 20 μg/rat of MeJA treatment

### 2.4 Surgery operation to embed the injection cannulas

The animals were first anesthetized with a mixture of ketamine and xylazine (100 and 2.5 mg/rat, respectively). Then, after fixing them with a stereotaxic apparatus, two 22‐gauge stainless steel guide cannulae were embedded bilaterally in the CA1 region consistent with the Paxinos and Watson Atlas of the rat brain (3.8 mm posterior to the bregma, 2.2 mm lateral from the midline, and 3.2 mm depth to the cortical surface). After that, two little screws and dental cement were utilized to fix the cannulas to the skullcap, followed by closing the cannulas with a stylet. To prevent infection and inflammation during the surgery and cannula implantation, all procedures were conducted in sterile conditions, following aseptic techniques, and using sterilized instruments. Moreover, in case of postoperative infections, both local (bacitracin ointment) and systemic (penicillin, 100,000 IU/kg) antibiotic treatments were prescribed (Geiger et al., 2008). After the surgery was performed, rats were individually maintained and allowed to recuperate for 1 week prior to drug treatments and behavioral tests.

### 2.5 Microinjection

The MeJA was initially dissolved in normal saline (0.9% NaCl) and injected into the brain's CA1 reign, 20 min prior to the induction of pain by Cap. The intra‐CA1 microinjection was conducted using a 27‐gauge needle and a 1 μL Hamilton microsyringe. The needle was inserted 1 mm beyond the tip of the guide cannula, and, in total, 2 μL of MeJA (1 μL for each side) was released in 1 min (Anaeigoudari et al., 2023; Hemati et al., 2021). After completion of each infusion, the needle remained for 30 s, and then it was drawn slowly back.

For clarification, the hippocampus is an essential part of the brain that plays a significant role in cognitive functions, such as memory and learning. Hence, in this study, MeJA was administered directly to the hippocampus to evaluate its specific impact on memory and learning processes that are controlled by the CA1 region. This method helps to deliver the compound in a more localized and controlled manner, minimizing its potential systemic effects or interactions with other areas of the brain (Heldt & Ressler, 2006).

### 2.6 Pain induction and assessment of pain behaviors

The orofacial pain‐representing behaviors of animals were evaluated following pain stimulation by injection of Cap. For this purpose, the rats were anesthetized by CO_2_, and their dental pulp was accessed by drilling a hole. Next, a sterile cotton piece soaked with 10 μL of Cap (100 μg/rat) was placed in the pulp cavity, followed by sealing the hole with dental cement. The rats were then set in observation chambers made of plexiglass. A mirror with an angle of 45° is embedded beneath the floor of the chamber, facilitating the unhindered rats monitoring. To assess pain‐related manners, the time of the typical pattern of face rubbing was considered. Each animal was monitored for 45 min, which was divided into nine 5‐min episodes. According to the Chidiac et al. scoring system (Clavelou et al., 1995; Dubuisson & Dennis, 1977), by measuring the time that each rat showed the below responses, a pain score was defined for each episode.

Calm, usual actions, such as grooming = 0; unusual head movements, such as shaking the head gently or continuously putting the jaw on the cage floor or wall = 1; persistent tremor of the lower jaw = 2; too much rubbing of the mouth with the leg, similar to consistent head grooming concentrated on the lower jaw = 3. Noteworthy, the behavioral actions were recorded using a video camera.

### 2.7 Morris water maze test

The orofacial pain impact on the animals’ learning and memory functions was studied by the Morris water maze (MWM) test, after assessing their pain behaviors (60 min after pain induction by Cap).

The water maze equipment includes a black cylindrical tank separated into four equal parts (quadrants) that are specified by the directions of N, E, S, and W. To evaluate the rats’ cognition performance, the tank was filled with water (20 ± 1°C) and 25 cm deep, and a covert platform was embedded in the center of one of the parts. Moreover, the walls of the test room were decorated with some geometric signs in such a way that were visible to rats. During the tests, a video camera located at the ceiling of the room recorded the animals’ movements and sent them to a computerized tracking system to analyze. It should be mentioned that, before the experimentation, the rats had 1 min to swim freely and become acquainted with the circumstances. The following describes the test procedure.

The acquisition/learning test procedure consisted of four blocks, each containing four trials. In each test, the rats were left swimming for 1 min to locate the concealed platform. They were allowed to remain for 30 s on it if they could locate the platform. If not, they were navigated to the platform by the experimenter. Moreover, the rats were given 5 min to rest between each trial and 20 min between each block. The average performance of rats to reach the platform, including their escape latency, the distance they traveled, and their swimming pace in four trials, were considered. Furthermore, the animals’ latency time, the traveled distance, and the swimming speed in all blocks—a total of 16 trials—were evaluated.

The animals’ spatial memory and their capabilities to retrieve the platform position were assessed using a prop test done 2 h after the last acquisition test (Kooshki, Abbasnejad, Esmaeili‐Mahani, et al., 2018). There was no platform in the maze for this test. Moreover, animals were released in the section opposite the target quadrant (where previously the platform was located) and given 1 min to swim independently. The whole time each animal spent in the target quadrant and its swimming route during the probe test were documented.

### 2.8 Gene expression

#### 2.8.1 RNA extraction and cDNA synthesis

To assess the changes in the level of gene expression, once the MWM test was performed, the rats’ hippocampus was dissected and homogenized. Total RNA was isolated using the RNX‐Plus solution (CinnaGen Company). Then, 30 μL of DEPC‐treated water was mixed with the obtained RNA, and its concentration was determined using a NanoDrop 2000/2000c spectrophotometer (PCRmax Lambda). Moreover, the extracted RNA was loaded on an agarose gel electrophoresis (1.5%) to confirm its quality. Thereafter, cDNA was synthesized utilizing Oligo‐dT primer and M‐MuLV reverse transcriptase enzyme (Fermentas), based on the manufacturer's instructions (Khaleghi & Khorrami, 2021).

#### 2.8.2 Real‐time polymerase chain reaction (RT‐PCR)

A Bio‐Rad iQ5 detection system (Bio‐Rad) was applied to assess the gene expression at the mRNA level using the cDNA related to each sample. This procedure was performed with Real Q Plus 2× Master Mix (Ampliqon). Moreover, the glyceraldehyde 3‐phosphate dehydrogenase gene was used as a housekeeping gene. The real‐time (RT) polymerase chain reaction (PCR) program was set as detailed below: 95°C for 15 min as the initial denaturation, followed by 40 RT PCR cycles that included the denaturation phase (20 s at 95°C), annealing phase (30 s at 60°C), and elongation phase (30 s at 72°C) (Dogani et al., 2021).

At the end of the process, the specificity of products and primer dimers was checked by considering the final melting curves. Moreover, by loading each prime‐pair product on agarose gel electrophoresis (1.5%), the accuracy of the process was evaluated. The primers related to the genes that were evaluated in this study are presented in Table 1 in detail. It is worth mentioning that all tests were done in duplicate, and their average was considered in calculating the relative mRNA expression levels based on the delta 2^−ΔΔ^ *^CT^* method.

**TABLE 1:** Primer sequences, real‐time polymerase chain reaction (RT‐PCR) fragment lengths, and NCBI accession numbers.

Primer name	Primer sequence	Size of PCR product	NCBI accession number
***TNF‐α***	F: ACCAGCAGATGGGCTGTACCTTAT R: ATGAAATGGCAAATCGGCTGACGG	107	NM_012675.3
***IL‐1β***	F: AAGACACGGGTTCCATGGTGAAGT R: TGGTACATCAGCACCTCTCAAGCA	97	NM_031512.2
***BDNF***	F: CGTGATCGAGGAGCTGTTGG R: CTGCTTCAGTTGGCCTTTCG	343	XM_008762078
***COX‐2***	F: CTCAGCCATGCA R: GGGTGGGCTTCAGCAGTAAT	172	NM_017232.4
***GAPDH***	F: GTCTTCACCACCACGGAGAAGGC R: ATGCCAGTGAGCTTCCCGTTCAGC	392	NM_017008

Abbreviations: GAPDH, glyceraldehyde 3‐phosphate dehydrogenase gene; IL‐1β, interleukin‐1β; TNF‐α, tumor necrosis factor‐α.

### 2.9 Statistical analysis

In order to statistically analyze these data, the SPSS software version 19 was used. The repeated‐measures analysis of variance (ANOVA) was applied to analyze the pain score and data obtained from four training blocks of the acquisition test. One‐way ANOVA was used to analyze the results of each block of hidden platform tests, probe training sessions, and RT‐PCR. Post hoc analysis was performed using the Tukey test, and the differences at *p* < .05 were considered significant. The data were presented as mean ± SEM.

## 3 RESULTS

### 3.1 Capsaicin‐induced pulpal nociception assay

**FIGURE 1:**
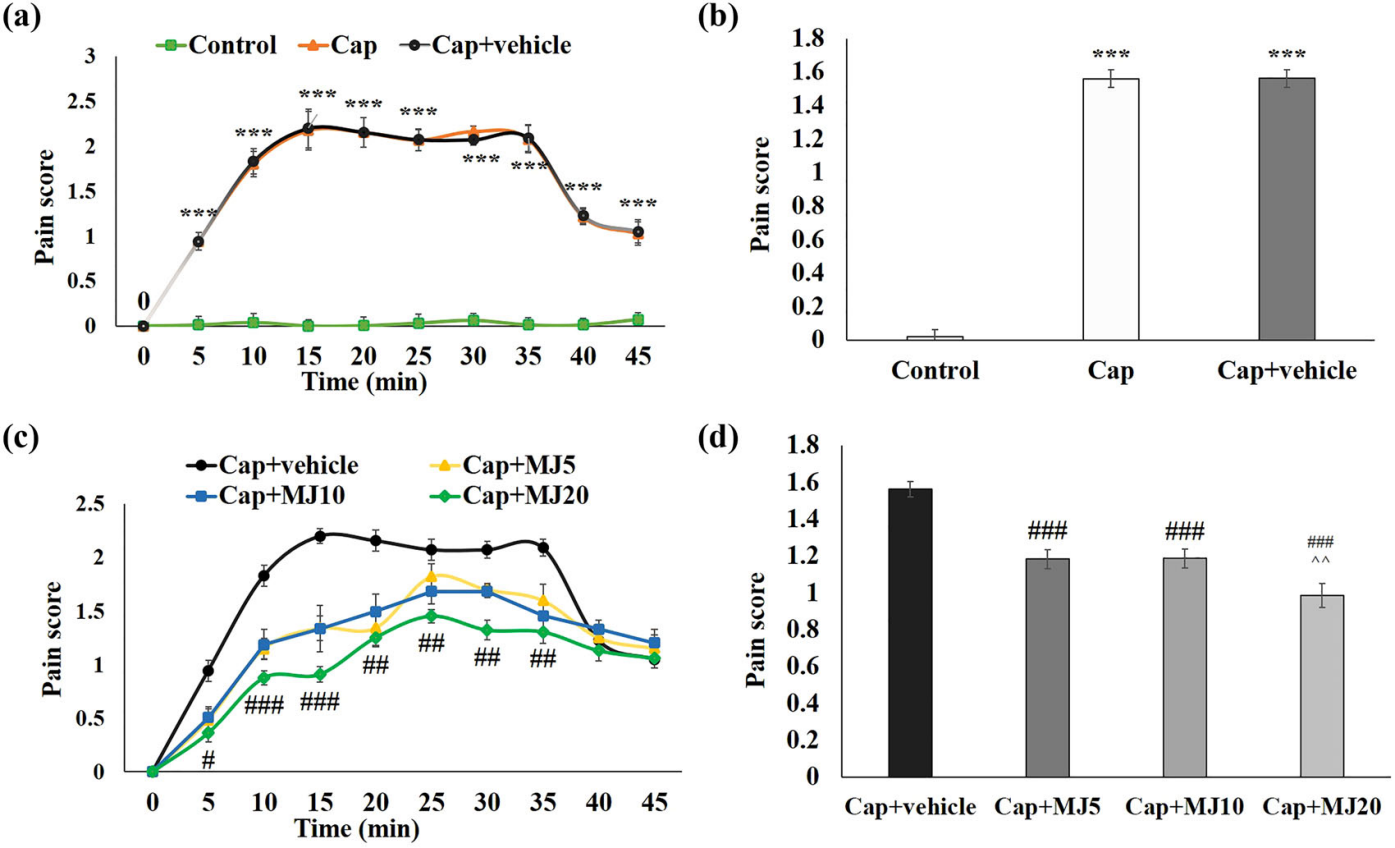
Effects of central administration of 5, 10, and 20 μg/rat of methyl jasmonate (MeJA) (MJ5, 10 and 20) on capsaicin (Cap)‐induced pulpal nociception. Parts (a) and (c) show time course of nociceptive behavior for each 5‐min block. Parts (b) and (d) show cumulative time of nociceptive behavior recorded over 45 min. Data are presented as mean ± SEM. Significant difference signs: ***p < .001 versus control group; ###*p* < .001, ##*p* < .01, and #*p* < .05 versus Cap + vehicle group; ^^*p* < .01 versus Cap + MJ5.

Cap was used to induce orofacial pain in the rats. As shown in Figure 1, Cap could significantly evoke nociceptive behavior as compared to the intact animals. Injection of MeJA considerably alleviated the animals’ pain behavior (*p* < .001). In both groups of Cap and vehicle, the pain score in the rats steadily increased until it reached its highest level (2.3 ± 0.3) after 15 min. This status continued for 15 min, and then it decreased gradually. However, pain scores in the animals that received MeJA were obviously decreased in the MeJA‐injected rats. The effect of MeJA was dose‐dependent. The pain score in the rats that received 20 μg MeJA was alleviated by 50% in comparison with Cap‐treated only (*p* < .001). Worth mentioning is that, in the MeJA‐treated rats, the pain level peaked much later and subsided more quickly compared to the control group.

### 3.2 Morris water maze

**FIGURE 2:**
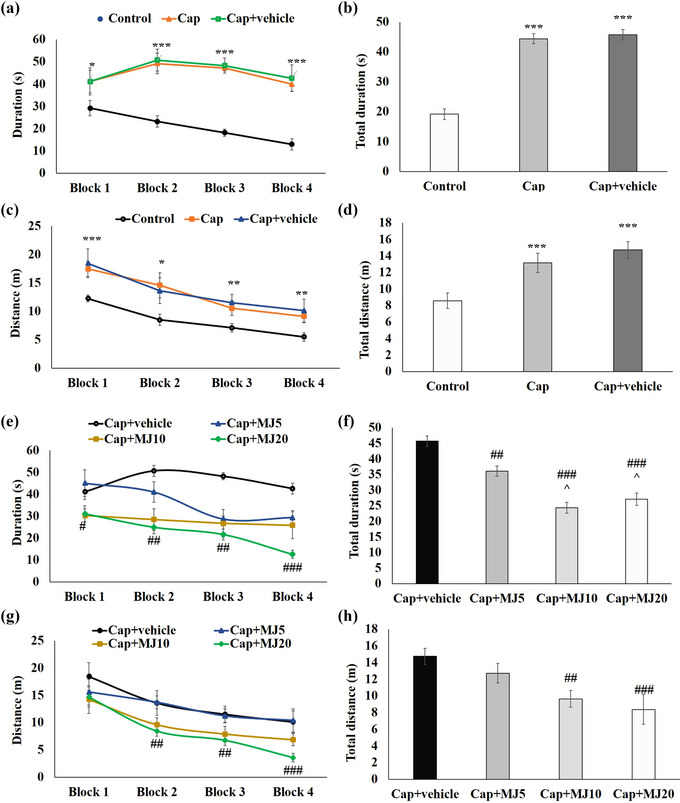
Effects of central administration of 5, 10, and 20 μg/rat of methyl jasmonate (MeJA) (MJ 5, 10 and 20) on duration and distance traveled (g and h) to reach the hidden platform in Morris water maze test. Data were presented as mean ± SEM. Significant difference signs: ***p < .001, **p < .01, and *p < .05 versus control group; ^###^ *p* < .001, ^##^ *p* < .01, and ^#^ *p* < .05 versus capsaicin (Cap) + vehicle group; ^*p* < .05 versus Cap + MJ5.

**FIGURE 3:**
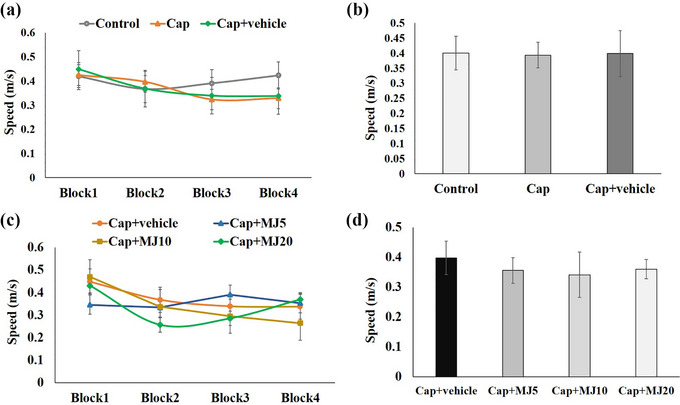
Effects of central administration of 5, 10, and 20 μg/rat of methyl jasmonate (MeJA) (MJ 5, 10 and 20) on speed (a‐d) of rats to reach the hidden platform in Morris water maze (MWM). Data were presented as mean ± SEM. Cap, capsaicin.

As shown in Figure 2a–h, there were significant differences in escape latency and the traveled distance across four blocks of acquisition between the intact and Cap‐injected groups. Cap‐induced pulpal nociception resulted in an increase in both latency and swimming distance to find the platform during acquisition trials (*p* < .001). The central administration of MeJA at 10 and 20 μg/rat significantly decreased the rats’ escape latency time and total distance traveled (*p* < .01). However, this treatment's impact on the swimming speed during acquisition trial blocks and total swimming speed was not significant among the groups (Figure 3).

**FIGURE 4:**
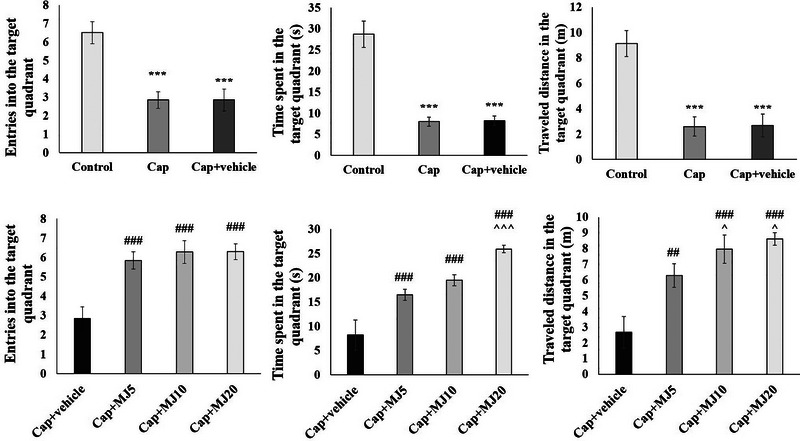
Effects of central administration of 5, 10, and 20 μg/rat of methyl jasmonate (MeJA) (MJ5, 10 and 20) on the results of acquisition of prob test in Morris water maze. Data were presented as mean ± SEM. Significant difference signs: ***p < .001, **p < .01, and *p < .05 versus control group; ^###^ *p* < .001, ^##^ *p* < .01, and ^#^ *p* < .05 versus capsaicin (Cap) + vehicle group; ^*p* < .05 versus Cap + MJ5.

Additionally, the results of prob tests indicated that the orofacial pain had a negative impact on the animals’ cognition performance. The rats experiencing nociception were less likely to enter the target quadrant, had lower distance traveled there, and shorter time spent in the area, as compared to the intact group. Though the administration of MeJA significantly ameliorated the impact of pain on the animals’ cognitive behaviors, the number of entries, distance traveled, and the time spent in the target quadrant were more than twice in the rats that received 10 and 20 μg of MeJA in comparison with Cap groups. Noteworthy, the MeJA activity was dose‐dependent, and consumption of 20 μg of this compound showed the best performance (Figure 4).

### 3.3 Gene expression

**FIGURE 5:**
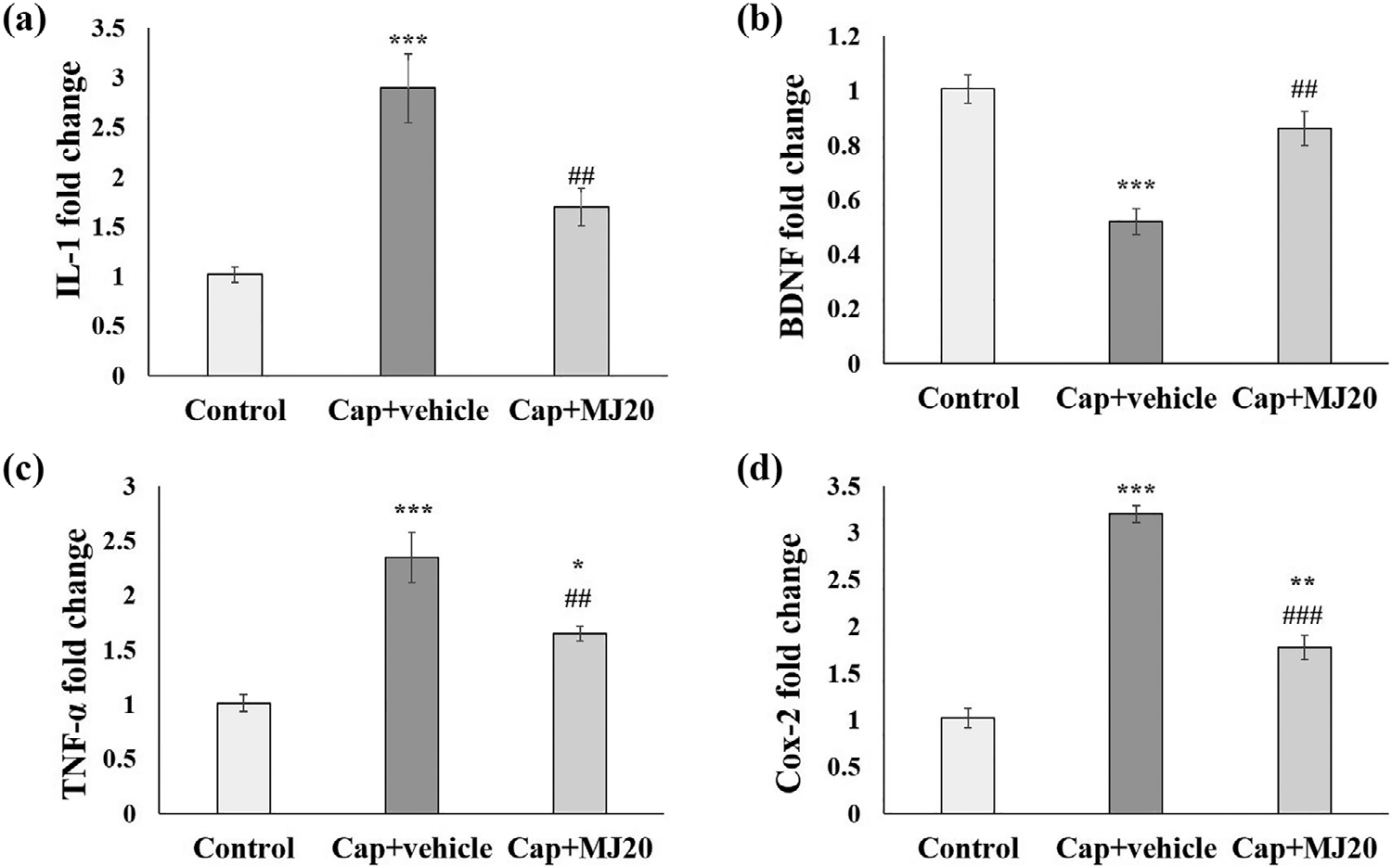
Effects of central administration of 20 μg/rat of methyl jasmonate (MeJA) (MJ20) on the gene expression levels of Interleukin‐1β (IL‐1β) (a), BDNF (b), tumor necrosis factor‐α (TNF‐α) (c), and Cox‐2 (d). Data were presented as mean ± SEM. Significant difference signs: ***p < .001, **p < .01, and *p < .05 versus control group; ^###^ *p* < .001, and ^##^ *p* < .01 versus capsaicin (Cap) + vehicle group; ^*p* < .05 versus Cap + MJ20.

To gain a deeper understanding of the effect of pain and MeJA on learning and memory functions, the expression levels of *TNF‐α*, *IL‐1β*, *COX‐2*, and *BDNF* genes were evaluated in rats’ hippocampus. The results showed that (Figure 5) the orofacial pain could reduce the BDNF expression markedly while increasing *TNF‐α*, *IL‐1β*, and *COX‐2* gene expression (*p* < .001). The levels of these genes in the pain‐experienced group were two to three times higher than in the control group. On the other hand, in the rats that received 20 μg of MeJA before pain induction, these features were considerably regulated and reversed to the control group level.

## 4 DISCUSSION

The current study aims to investigate the effects of orofacial pain on memory and learning. In addition, it was studied whether the central administration of MeJA into the animals’ hippocampus could alleviate the pain's detrimental effects on cognition functions or not. The hippocampus is a part of the brain involved in learning, memory, and emotions. It is also susceptible to inflammation and plays a critical role in pain processing (McCarberg & Peppin, 2019). Hence, selecting the hippocampus as the site for administering MeJA and studying the expression of inflammatory genes can help us understand the influence of inflammation on pain‐related behaviors and the molecular mechanisms involved in pain perception and modulation in this brain region. Our findings suggested that Cap‐induced orofacial pain could considerably impair the animals learning and memory, whereas these impairments were significantly inhibited by MeJA treatment. This improvement is most likely due to the modulating effect of the MeJA on the expression of inflammatory genes in the hippocampus.

The utilization of Cap to induce trigeminal pain in rats is considered a conventional laboratory model to investigate the potential mechanism(s) involved in orofacial distress. The interaction of Cap with TRPV1 receptors expressed in dental pulp causes the overproduction of molecules mediating nociceptive and inflammatory markers, such as IL‐1β and TNF‐α. Both IL‐1β and TNF‐α promoted COX‐2 protein expression and the subsequent production of the final inflammatory mediator, prostaglandins (Barreto et al., 2016). Cap stimulates pulpal nociception via increasing COX‐2 expression in the critical brain center and receiving preferentially nociceptive afferent signals from the orofacial area (Kooshki, Abbasnejad, Mahani, et al., 2018; Mohamadi‐Jorjafki et al., 2020). It seems that COX‐2 has an important role in the pathophysiology of inflammatory pain in the orofacial regions. Triggering TRPV1 can enhance the synaptic transmission at Vc second‐order neurons. Furthermore, Cap modifies the sensitivity of neurons, facilitating the use of additional stimulators (Kooshki, Abbasnejad, Esmaeili‐Mahani, et al., 2018).

Noteworthy, the overexpression of IL‐1, TNF‐α, and IL‐6 mRNA has previously been reported in the hippocampal and cortical brain regions of mice experiencing cognitive diseases (Sun et al., 2022; Wang et al., 2023). Given that hippocampus is responsible for cognition performance, particularly memory and learning (Olsen et al., 2012), increasing these inflammatory factors in the hippocampus following pain induction can impair these cognitive functions.

Pain‐associated cognitive dysfunction has already been examined in numerous rodent models of pain (Kooshki et al., 2016). However, studying orofacial pain and its association with learning and memory dysfunction has recently come into focus for researchers. In this regard, we found that the orofacial pain‐suffered rats had difficulty learning and reminding the platform location in the MWM test. These pain side effects can be attributed to the loss of neuronal cells’ function in the hippocampal tissue (Baghishani et al., 2018).

In a study conducted by Hemati et al., the effects of three doses of MeJA (0.5, 2.5, and 5 μg/rat) on learning and memory, anxiety‐like behaviors, and brain oxidative stress were investigated. The data showed that MeJA, dose‐dependently, could improve passive avoidance learning and memory and reduce anxiogenic behaviors. Furthermore, central microinjection of MeJA significantly decreased hydrogen peroxide concentration, and increased reactive oxygen species scavenger activity (catalase and peroxide enzymes) in rats’ hippocampus as well as prefrontal cortex (Hemati et al., 2021).

In another study, the efficacy of three doses of MeJA (2.5, 5, and 10 μg/rat) on food‐related behaviors in rats was evaluated. The results indicated the potential of MeJA to modulate feeding‐related behavior and Orx1R expression in the hypothalamus of rats (Anaeigoudari et al., 2023).

It has been previously reported that pain can induce apoptosis in the brain cells (Kooshki et al., 2017). We found that MeJA treatment leads to a considerable increase in the BDNF gene levels while decreasing *TNF‐α*, *IL‐1β*, and *COX‐2* levels. According to the previous in vitro and in vivo studies, BDNF is involved in the anti‐inflammatory and anti‐apoptotic pathways in the hippocampus (Xu et al., 2017). In contrast, the other three factors support the inflammatory process. For instance, IL‐1β is known as a robust pro‐inflammatory cytokine that can activate microglia and raise the permeability of the blood–brain barrier, encouraging leukocyte infiltration and upregulation of other pro‐inflammatory molecules, such as PGE2, COX2, and TNFα (Laflamme et al., 1999; Ziebell & Morganti‐Kossmann, 2010). Particularly, COX2 shows basal overexpression in the brain and plays a substantial role in brain inflammations (Kooshki et al., 2016; Singh et al., 2020).

According to our results, the hippocampal levels of BDNF considerably decreased in the rats that experienced pain. These results are in agreement with previous studies. A study by Duric and McCarson (2006) showed the impaired spatial learning and memory induced by neuropathic pain. They also reported decreased BDNF levels in the hippocampus of rats (Duric & McCarson, 2006). In a recent study, the memory dysfunction induced by trigeminal neuralgia was related to a reduction in the activities of the c‐AMP‐responsive element binding protein (CREB)/BDNF pathway, which plays a substantial role in the remodeling of synapses and consolidation of memory (Kooshki, Abbasnejad, Mahani, et al., 2018). It has been demonstrated that the reduction of neurotrophic factors (such as BDNF) levels and their activities can degrade the plasticity of synapses and impair cognitive functions in some pathological situations, like inflammatory diseases (Lima Giacobbo et al., 2019; Xie & Yung, 2012).

Increasing the oxidative markers can also induce inflammation and interrupt neurobehavioral functions, such as learning and memory (Bhatt et al., 2018; Khorrami et al., 2023; Mohseni‐Moghaddam et al., 2022). However, antioxidant agents owing to their free radical scavenging properties can ameliorate cognitive impairments and pain‐trigger sensitivity toward neuroinflammatory conditions (Kaur et al., 2020; Khorrami et al., 2019). As reported, MeJA is a strong antioxidant (Reyes‐Díaz et al., 2016) and could decrease hippocampal and striatal levels of malondialdehyde, which is a well‐known oxidative stress indicator (Aluko et al., 2021; Hashim, 2011). Likewise, it has been shown that intraperitoneal administration of MeJA boosted levels of choline acetyltransferase and antioxidant enzymes in the brain cortex (Cacabelos et al., 2016). It seems that the beneficial MeJA on pain‐induced learning and memory impairment may be performed by its antioxidant properties. However, this issue needs to be investigated by further studies. Furthermore, in agreement with our results. It has been recently reported that MeJA can modulate monoaminergic neurotransmission, antioxidant system, and Nrf2 expressions and alleviate memory dysfunctions caused by chronic stress (Aluko & Umukoro, 2020). The ability of MeJA to repel mouse parkinsonian‐like symptoms has also been reported (Alabi et al., 2019). Likewise, findings of a newly published investigation indicate the neuroprotective, antioxidant, and neuron‐regenerative properties of this compound (Omayone et al., 2023).

It has been clear that pain acts as a disruption of central cholinergic neurotransmission and therefore impairs cognitive functions (Ardeshiri et al., 2017; Erfanparast et al., 2017). However, the precise mechanism through which MeJA plays such role is still unknown. The results of recent studies suggest that MeJA may reduce pain‐associated memory damage by inhibiting oxidative stress processes and increasing cholinergic neurotransmission (Eduviere et al., 2015).

Considering the fact that pain can cause cognitive deficiencies mediated by increased levels of inflammatory proteins, it could be concluded that MeJA, by the inhibition of pro‐inflammatory factors and increasing the anti‐inflammatory agents such as BDNF, ameliorates the pain‐induced dysfunctions in rats.

## AUTHOR CONTRIBUTIONS

**Fatemeh Mohammadinia**: Methodology; investigation; writing—original draft. **Saeed Esmaeili‐Mahani**: Conceptualization; resources; supervision; writing—review and editing. **Mehdi Abbasnejad**: Writing—review and editing; supervision; resources. **Manijeh Dogani**: Methodology; formal analysis. **Ali Mohammad Poorrahimi**: Validation.

## CONFLICT OF INTEREST STATEMENT

The authors declare no conflict of interest.

## FUNDING INFORMATION

This research did not receive any specific grant from funding agencies in the public, commercial, or not‐for‐profit sectors.

### PEER REVIEW

The peer review history for this article is available at https://publons.com/publon/10.1002/brb3.3502.

## Data Availability

The datasets generated during and/or analyzed during the current study are available from the corresponding author on reasonable request.
